# Gene silencing in tick cell lines using small interfering or long double-stranded RNA

**DOI:** 10.1007/s10493-012-9598-x

**Published:** 2012-07-07

**Authors:** Gerald Barry, Pilar Alberdi, Esther Schnettler, Sabine Weisheit, Alain Kohl, John K. Fazakerley, Lesley Bell-Sakyi

**Affiliations:** 1 The Roslin Institute and Royal (Dick) School of Veterinary Studies, University of Edinburgh, Easter Bush, Midlothian EH25 9RG UK; 2 Present Address: Institute of Infection, Immunity and Inflammation, MRC-University of Glasgow Centre for Virus Research, 464 Bearsden Road, Glasgow, G61 1QH UK; 3 Present Address: MRC-University of Glasgow Centre for Virus Research, 8 Church Street, Glasgow, G11 5JR UK; 4 Present Address: Institute for Animal Health, Pirbright Laboratory, Ash Road, Pirbright, Surrey GU24 0NF UK

**Keywords:** Tick cell line, Gene silencing, RNAi, Transfection, dsRNA, siRNA

## Abstract

Gene silencing by RNA interference (RNAi) is an important research tool in many areas of biology. To effectively harness the power of this technique in order to explore tick functional genomics and tick-microorganism interactions, optimised parameters for RNAi-mediated gene silencing in tick cells need to be established. Ten cell lines from four economically important ixodid tick genera (*Amblyomma, Hyalomma, Ixodes* and *Rhipicephalus* including the sub-species *Boophilus*) were used to examine key parameters including small interfering RNA (siRNA), double stranded RNA (dsRNA), transfection reagent and incubation time for silencing virus reporter and endogenous tick genes. Transfection reagents were essential for the uptake of siRNA whereas long dsRNA alone was taken up by most tick cell lines. Significant virus reporter protein knockdown was achieved using either siRNA or dsRNA in all the cell lines tested. Optimum conditions varied according to the cell line. Consistency between replicates and duration of incubation with dsRNA were addressed for two *Ixodes scapularis* cell lines; IDE8 supported more consistent and effective silencing of the endogenous gene subolesin than ISE6, and highly significant knockdown of the endogenous gene 2I1F6 in IDE8 cells was achieved within 48 h incubation with dsRNA. In summary, this study shows that gene silencing by RNAi in tick cell lines is generally more efficient with dsRNA than with siRNA but results vary between cell lines and optimal parameters need to be determined for each experimental system.

## Introduction

Ticks are haematophagous arthropods that target a wide range of terrestrial vertebrates. While ticks can cause harm to their hosts directly through skin damage and blood loss, they also transmit numerous bacteria, viruses and protozoa (Jongejan and Uilenberg 2004). Many of these are pathogenic for humans and/or domestic animals. Acaricide treatment and vaccination have been two main strategies for protection against ticks and tick-borne diseases. Although these measures have helped greatly, resistance to acaricide treatment is increasing, while vaccination is often impractical or insufficiently effective (George et al. 2004; Willadsen 2006; Shkap et al. 2007; de la Fuente et al. 2007a; Latif and Hove 2011). Progress in the development of vaccines and alternative tick control methods has been aided by the availability of complete or partial genome sequence data for many of the tick-borne pathogens and for some tick species such as *Ixodes scapularis, Rhipicephalus (Boophilus) microplus* and *Amblyomma variegatum* (Nene 2009). Concurrent development of molecular biological tools and techniques such as RNA interference (RNAi), proteomics and transcriptomic analysis is greatly facilitating research into tick-host-pathogen interactions.

In numerous fields of research, the use of cell lines has allowed the discovery of many aspects of immunity and cell biology. Progression with the establishment and use of tick cell lines is improving and adding pace to multiple aspects of tick and tick-borne pathogen biology research (Bell-Sakyi et al. 2007, 2011). The knockdown of protein expression by RNAi is a powerful tool that can be used to investigate gene function in ticks (Kocan et al. 2007; Nijhof et al. 2007; de la Fuente et al. 2007c; Kurscheid et al. 2009; Barnard et al. 2012). Introduction of long double stranded RNA (dsRNA) into whole ticks can be achieved by inoculation, artificial feeding or immersion (de la Fuente et al. 2007c). The silencing can be relatively long-term, with the effect carried on from the female tick into the next generation of eggs and larvae (Kocan et al. 2007; Nijhof et al. 2007). RNAi is also effective in tick cell lines (Blouin et al. 2008; de la Fuente et al. 2007b), although the efficiency and consistency of gene silencing is generally poor (Jose de la Fuente, personal communication and authors’ unpublished results). RNAi has been used to investigate the function of different tick genes that are involved in various aspects of tick biology (de la Fuente et al. 2007b, c; Kurscheid et al. 2009). However, in vitro silencing has been reported in cell lines derived from only three tick species: *I. scapularis* (de la Fuente et al. 2007b; Blouin et al. 2008), *Ixodes ricinus* (Pedra et al. 2010) and *R.* (*B.*) *microplus* (Kurscheid et al. 2009; Zivkovic et al. 2010a). There is a need to optimise protocols for the use of long dsRNA in a range of cell lines derived from ticks of medical and veterinary importance, to enhance research into tick functional genomics and tick-host-pathogen interactions.

The use of small interfering RNA (siRNA) to silence protein expression in ticks or tick cell lines has been quite limited. Narasimhan et al. (2007) achieved a reduction in expression of the *I. scapularis* salivary protein *salp25D,* and consequent reduction in acquisition of *Borrelia burgdorferi* infection, in ticks inoculated with siRNAs specific to *salp25D*. Pedra et al. (2010) silenced fucosyl transferase protein expression in the *I. ricinus* cell line IRE/CTVM19 with resultant reduction in *Anaplasma phagocytophilum* infection levels. As with long dsRNA, there is a need to develop and optimise protocols for the use of siRNA in tick cell lines.

The effectiveness of long dsRNA in *I. scapularis* cell lines has previously been examined in studies conducted by Kurtti et al. (2008) in which ISE6 cells expressing a red fluorescent protein, dsRed, were transfected with dsRNA targeting the dsRed expression, and Blouin et al. (2008) who used Cy3 labelled dsRNA to monitor uptake in IDE8 cells. In the present study, we have utilised RNAi to examine some of the parameters that affect efficient knockdown of mRNA levels and protein expression in tick cell lines. Multiple transfection reagents were screened against a panel of tick cell lines to establish siRNA and dsRNA transfection efficiencies for each combination of cell line and reagent. The mosquito-borne alphavirus Semliki Forest virus (SFV) expressing *Renilla* luciferase (*RLuc*) was used as a model system (Kiiver et al. 2008; Fragkoudis et al. 2009b) to test the efficiency of silencing a virus-expressed protein in infected tick cells using long dsRNA or siRNA. The knockdown of endogenous gene mRNA transcript levels using long dsRNA was then measured in two *I. scapularis* cell lines. It was found that both siRNAs and long dsRNA can be used effectively in tick cell lines, but different conditions are required for each, while the nature of each particular cell line also plays an important role.

## Materials and methods

### Tick cell lines

Ten cell lines representing four ixodid tick genera of medical and veterinary importance (Table 1) were grown in L-15 (Leibovitz)-based culture media supplemented with foetal bovine serum (FBS) at 28 or 32 °C. The cell lines were provided by the Tick Cell Biobank (http://tickcells.roslin.ac.uk).

**Table 1:** Tick cell lines used in the study

Tick species	Vector of:	Cell line	Reference
*Amblyomma variegatum*	*Ehrlichia ruminantium*, *Rickettsia africae*, *Theileria mutans, Theileria velifera,* Dugbe virus	AVL/CTVM13	Bell-Sakyi et al. (2000)
*Hyalomma anatolicum*	*Theileria annulata, Theileria lestoquardi,* Crimean-Congo haemorrhagic fever virus	HAE/CTVM9	Bell-Sakyi (1991)
*Ixodes ricinus*	*Borrelia* spp., *A. phagocytophilum*, *Babesia divergens*, tick-borne encephalitis virus, louping ill virus	IRE/CTVM19	Bell-Sakyi et al. (2007)
*Ixodes scapularis*	*Borrelia burgdorferi*, *A. phagocytophilum, Babesia microti*, deer tick virus	IDE8	Munderloh et al. (1994)
		ISE6	Kurtti et al. (1996)
*Rhipicephalus* (*Boophilus*) *decoloratus*	*Anaplasma marginale*, *Babesia bigemina*	BDE/CTVM16	Bell-Sakyi (2004)
*Rhipicephalus* (*Boophilus*) *microplus*	*Anaplasma marginale*, *Babesia bigemina*, *Babesia bovis*	BME/CTVM23	Alberdi et al. in press
*Rhipicephalus appendiculatus*	*Theileria parva*, Nairobi sheep disease virus	RA257*	Varma et al. (1975)
		RAE/CTVM1*	Bell-Sakyi (2004)
*Rhipicephalus evertsi*	*Anaplasma marginale, Babesia caballi*, *Theileria equi*	REE/CTVM28	Unpublished**

* Either RA257 or RAE/CTVM1 was used in experiments with *R. appendiculatus* cells

** REE/CTVM28 was derived by a standard technique from embryos of a South African strain of *R. evertsi* (Nijhof et al. 2010) and was grown at 28 °C in L15/MEM medium (Bell-Sakyi 2004). The parent *R. evertsi* ticks were kindly provided by Dr. Ard Nijhof and Prof. Frans Jongejan, Utrecht Centre for Tick-borne Diseases, Utrecht University, The Netherlands

### Virus strain used and infection protocol

A strain of SFV expressing *Renilla* luciferase (*RLuc*) designated SFV4-st*RLuc,* was used (Kiiver et al. 2008). This virus was made as previously described (Liljestrom et al. 1991) from a plasmid kindly provided by Professor Andres Merits, University of Tartu, Estonia. The *RLuc* gene is located in the structural region of the genome between the capsid and envelope genes. The foreign protein is cleaved from the capsid by the capsid autoprotease and from the envelope glycoproteins by the presence of a self-processing foot-and-mouth disease virus 2A peptide. To infect cells, the virus was diluted in phosphate buffered saline containing 0.75 % bovine serum albumin (PBSA) and 50 μl of this suspension was then added to the cells at a multiplicity of infection (MOI) of 3.

### Transfection of tick cells

The transfection reagents Lipofectamine 2000 (Invitrogen), XtremeGENE siRNA transfection reagent (Roche), DharmaFECT 1 (Thermo Scientific), HiPerFect (Qiagen), Lipofectamine RNAiMAX (Invitrogen) and siPORT Amine transfection reagent (Ambion) were tested. Cells for transfection and RNAi experiments were seeded 24 h prior to use at 0.7–1.0 × 10^6^ cells/ml in either sealed flat-sided culture tubes (Nunc; 2 ml total volume per tube) or 24 well plates (Nunc; 1 ml total volume per well) and incubated in ambient air at 28 °C. The dsRNA (400 ng per tube or 200 ng per well of cells) or siRNA (50 nM concentration) was first incubated at room temperature in OptiMEM (OptiMEM + glutamax, Invitrogen, 50 μl per tube or well of cells to be transfected) for 5 min and then mixed with the transfection reagent (1 or 2 μl of transfection reagent (siRNA or dsRNA respectively) + 49 μl OptiMEM) and incubated for 20 min at room temperature. OptiMEM alone was used if no transfection reagent was required. Immediately before addition of test reagents, the volume of culture medium in each tube or well was reduced by half. Aliquots of 100 μl of incubated transfection mix were added to the medium in each tube or well and the cells were incubated at 28 °C for 24 h. Fresh complete culture medium was then added to restore the original volume if longer incubation periods were required.

### Preparation of dsRNA

To make long dsRNA (approximately 600 bp) Ambion’s MEGAscript RNAi Kit was used. Gene-specific primers linked with T7 promoter regions were used to amplify regions of the *RLuc* gene from the SFV4-st*RLuc* genome, the endogenous gene subolesin (Blouin et al. 2008) from the *I. scapularis* cell line ISE6 and the endogenous gene 2I1F6 (de la Fuente et al. 2007b) from the *I. scapularis* cell line IDE8. The PCR products were in vitro transcribed and allowed to anneal to form dsRNA before purification and quantification. Fluorescent long dsRNA was synthesised using a fluorescein RNA labelling mix (Roche) following the manufacturer’s instructions. dsRNA targeting enhanced green fluorescent protein (eGFP) was used as a negative control. The primers and PCR conditions used are listed in Table 2.

**Table 2:** List of primer sequences used in the study

	Upstream/downstream primer sequences (5′–3′)	PCR annealing conditions
Gene for dsRNA		
*Renilla* Luciferase	**TAATACGACTCACTATAGGG**ATGACTTCGAAAGTTTATGATCCAG/**TAATACGACTCACTATAGGG**CTGCAAATTCTTCTGGT TCTAACTTTC	60 °C, 30 s
eGFP	**TAATACGACTCACTATAGGG**ATGGTGAGCAAGGGCGAGGAGCTGTTC/**TAATACGACTCACTATAGGG**CTGGGTGCTCAGGTAGTGGTTGTCGGGC	60 °C, 30 s
*I. scapularis* 2I1F6	**TAATACGACTCACTATAGGG**CAACCCCAAGATCGTCAACT/**TAATACGACTCACTATAGGG**ACGCGTCCTTACGTTTCACT	58 °C, 30 s
*I. scapularis* subolesin	**TAATACGACTCACTATAGGG**TACTATGGCTTGCGCAACATTAAAG/**TAATACGACTCACTATAGGG**TACTTTATGACAAATAGCTTGGAG	
Gene for real-time PCR		
*I. scapularis* 2I1F6	GAATTCGAGCGTGGACCTTA/ATTGTCTCCGCACTCTTCGT	53 °C, 30 s
*I. scapularis* subolesin	AGCAGCTCTGCTTGTCGTCT/TCGTACTCGTCGCGTATCTG	54 °C, 30 s
*I. scapularis* β-actin	AAGGACCTGTACGCCAACAC/ACATCTGCTGGAAGGTGGAC	53 °C, 30 s

The T7 promoter region is indicated in bold type

### Preparation of siRNA

Long dsRNAs were made as described above and then Shortcut RNase III (New England Biolabs) was used to cleave the long dsRNA into a heterogeneous pool of siRNAs. This cleavage was confirmed by gel electrophoresis. Block-it green fluorescent siRNA (Invitrogen) was used to analyse transfection efficiency. Scrambled, non-targeting siRNA (Thermo Scientific) was used as a negative control.

### Silencing of *RLuc* expression in SFV-infected tick cell lines

Cells were transfected with siRNA or dsRNA as described above and were then infected with SFV4-st*RLuc* virus 24 h later. The cultures were incubated for a further 48 h before *RLuc* production was measured by luciferase assay as follows. Equal numbers of cells from each replicate of each cell line to be assayed were isolated by centrifugation and lysed with a passive lysis buffer (Promega). *RLuc* expression levels were assessed using a Luciferase reporter assay system (Promega) and a GloMax Luminometer (Promega) following the manufacturer’s protocols.

### Silencing of endogenous tick genes

Cells were transfected with long dsRNA as described above and incubated for 24–96 h. RNA was then extracted from the cell cultures using an RNeasy kit (Qiagen). Equal amounts of RNA were reverse transcribed into cDNA and then real-time PCR using gene-specific primers was used to quantify levels of RNA transcripts. The housekeeping gene β-actin was used as a reference control gene. The primers and conditions used for the real-time PCR are listed in Table 2.

### Microscopy

For fluorescence and light microscopy, a Zeiss Axiovert inverted microscope with observer D1 was used. The photomicrographs were taken and analysed using Zeiss Axiovision software. For each cell line a minimum of 400 cells were counted at 100× magnification with simultaneous normal transmitted light and UV reflected light 24 h post-transfection. Confocal microscopy was carried out using a Zeiss LSM710 confocal microscope with Zeiss Zen software.

### Statistical analysis

The unpaired two-sample *t* test was used to determine significant differences between groups in experiments involving measurement of luciferase activity or real-time PCR. A *p* value of <0.05 was considered significant.

## Results

### Selection and efficiency of transfection reagents

**Fig. 1:**
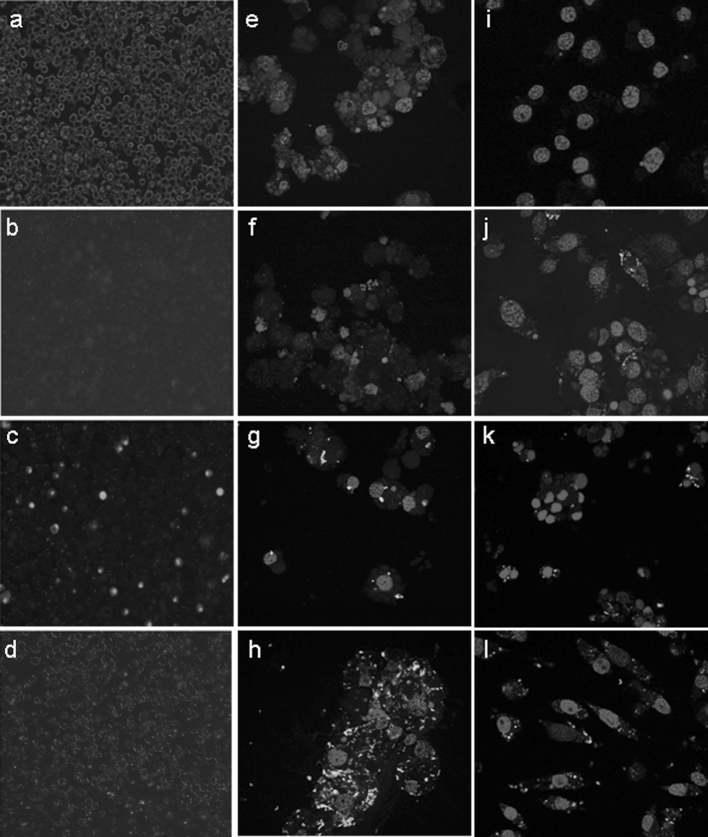
Images of tick cells 24 h after addition of fluorescent siRNA (**a**–**h**) or dsRNA (**i**–**l**) in the presence or absence of a transfection reagent. **a**–**d** photomicrographs of BDE/CTVM16 cells taken at ×100 magnification with simultaneous normal transmitted light and UV reflected light to facilitate counting of fluorescent (*green* siRNA) and non-fluorescent cells. **e**–**h** confocal images of IRE/CTVM19 cells taken at ×630 magnification; cell nuclei are stained *blue*, while the siRNAs are *green*. **i**–**l** confocal images of IDE8 cells taken at ×630 magnification; cell nuclei are stained *blue*, while the long dsRNAs are *green*. **a**, **e**, **i** show untreated control cells; **b**, **f**, **j** show cells to which siRNA or dsRNA alone was added; **c**, **g**, **k** show cells to which siRNA or long dsRNA mixed with Lipofectamine 2000 was added; **d**, **h**, **l** show cells to which siRNA or long dsRNA mixed with Xtreme was added. (Color figure online)

Generally, at least in mammalian cells, siRNAs are delivered with a transfection reagent such as liposomes. To investigate whether transfection reagents are required for tick cell lines and to determine transfection efficiency and toxicity, six different transfection reagents were tested on two tick cell lines, BDE/CTVM16 and IRE/CTVM19. Both cell lines grow as single cells rather than connected cell sheets and are therefore easy to count visually. The cells were incubated alone or with green fluorescent siRNA in the presence or absence of a transfection reagent and the number of green cells in each culture was quantified by fluorescence microscopy (minimum 400 cells counted); Fig. 1a–d shows low power images of BDE/CTVM16 cells used for counting. All cultures appeared healthy 24 h post-transfection but only two transfection reagents resulted in fluorescence of >10 % of the cells. These were Lipofectamine 2000 and XtremeGENE (hereafter referred to as Xtreme). Figure 1e–h shows the appearance, by confocal microscopy, of IRE/CTVM19 cells 24 h after transfection with the fluorescent siRNA. Interestingly, fluorescence was confined to foci within the cytoplasm. These cultures were monitored for a further 13 days. BDE/CTVM16 cells lost fluorescence by 11 days post-transfection while only a few fluorescent IRE/CTVM19 cells were still detectable at day 14 post-transfection. No fluorescent cells were visible in the absence of a transfection reagent.

Following the same protocol, Lipofectamine 2000 and Xtreme were tested on a further seven cell lines. Presence of fluorescence was quantified by microscopy (Table 3). Overall, Xtreme resulted in higher numbers of detectably transfected, i.e., fluorescent, cells for all cell lines tested except for the *R.* (*B.*) *microplus* line BME/CTVM23. There was, however, wide variation in transfection efficiency between cell lines with both reagents. For example, low transfection levels were achieved with IDE8 and ISE6 cells with both reagents while IRE/CTVM19 cells showed low levels of transfection with Lipofectamine 2000 but high levels with Xtreme. By day 4, the proportion of positive cells in all cultures had decreased by 10–30 %; by day 14 no green cells were present in HAE/CTVM9 and IDE8 for both reagents and RA257 for Lipofectamine 2000, while green cells remained until day 28, albeit in decreasing numbers, in AVL/CTVM13, BME/CTVM23 and RA257 transfected with Xtreme. It was also noticeable that Xtreme had little apparent toxic effect on the cells, while over time treatment with Lipofectamine 2000 adversely affected the cells, especially after the first week of treatment.

**Table 3:** Efficiency of uptake of fluorescent siRNA by nine tick cell lines

Cell line	Percentage of cells containing detectable green fluorescence when incubated with siRNA and:		
	Lipofectamine 2000	Xtreme	No reagent
AVL/CTVM13	34	40	0
BDE/CTVM16	40	56	0
BME/CTVM23	57	41	0
HAE/CTVM9	16	42	0
IRE/CTVM19	19	67	0
IDE8	20	31	0
ISE6	23	33	0
RA257	40	66	0
REE/CTVM28	>20	ND	0

Cells were incubated with fluorescent siRNA either alone or with one of two transfection reagents, Lipofectamine 2000 or Xtreme (one tube of cells per treatment). The data for the REE/CTVM28 cell line transfected with Lipofectamine 2000 is an estimate as it is not possible to distinguish individual cells in these cultures. *ND* not done

### Parameters for silencing a virus reporter protein in tick cell lines using siRNA

**Fig. 2:**
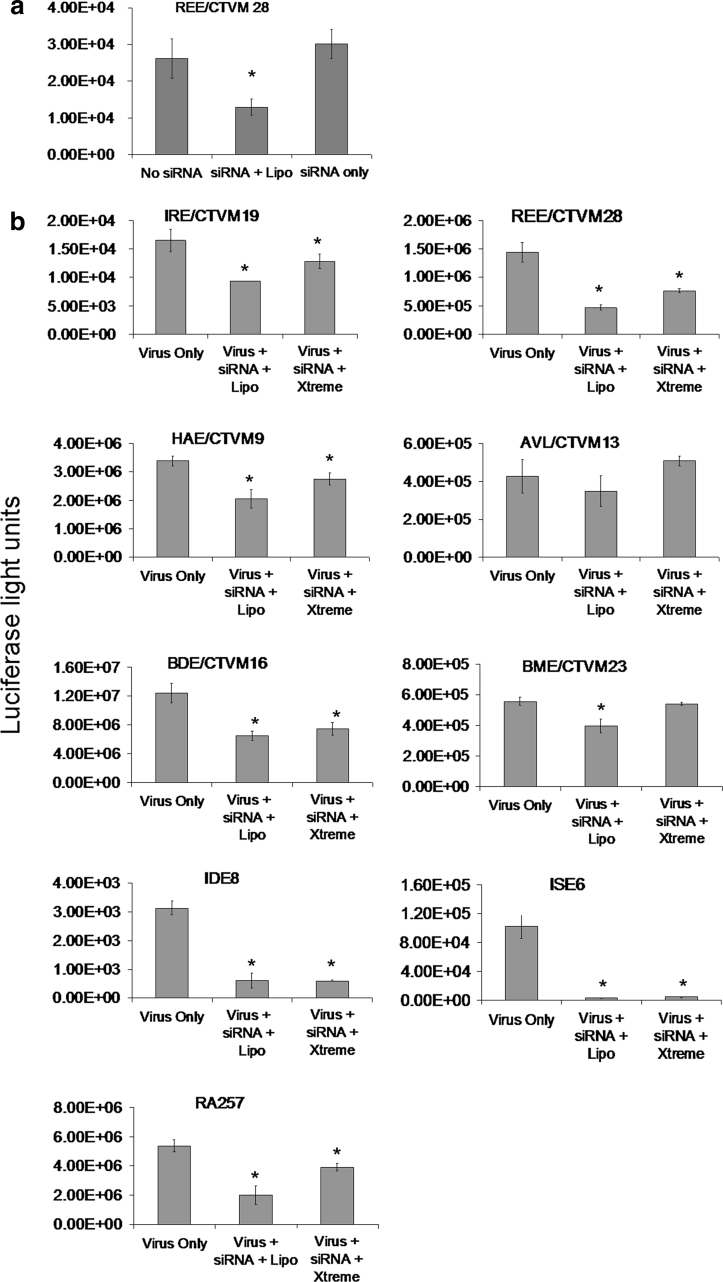
Silencing of a virus reporter protein in tick cells by siRNA treatment. **a** REE/CTVM28 cells were incubated with siRNA against *RLuc* in the presence or absence of Lipofectamine 2000 (Lipo) and then infected 24 h later with SFV expressing *RLuc*. Luciferase levels (*y-*axis) were then measured 48 h later in cultures with SFV alone (Virus only), siRNA with Lipofectamine 2000 followed by SFV (Virus + siRNA + Lipo) and siRNA alone followed by SFV (Virus + siRNA only). The values shown are means of three replicate cultures and the *error bars* are standard deviations of the mean. * Significant decrease from the virus only control. **b** Nine tick cell lines were incubated with siRNA targeting *RLuc* in the presence of a transfection reagent and then infected 24 h later with SFV expressing *RLuc*. Luciferase levels (*y*-axis) were then measured 48 h later in cultures with SFV alone (Virus only), siRNA with Lipofectamine 2000 followed by SFV (Lipo + siRNA + virus) and siRNA with Xtreme followed by SFV (Xtreme + siRNA + virus). The values shown are means of four replicate cultures and the *error bars* are standard deviations of the mean. The scale used for each *y*-axis reflects the range of luciferase levels generated in the particular tick cell line. * Significant decrease from the virus only control

The alphavirus SFV is not known to be transmitted by ticks; it is however capable of infecting tick cells in vitro (Bell-Sakyi et al. 2011; Garcia et al. 2005). The virus construct SFV4-st*RLuc* has been engineered to express *RLuc* when it infects cells (Kiiver et al. 2008), providing a sensitive and rapidly-measurable indication of virus gene expression. As a pilot experiment, the REE/CTVM28 cell line was used to examine the possibility that siRNA treatment could be effective without a transfection reagent. When fluorescent siRNA was added to these cells with Lipofectamine 2000, approximately 20 % of cells displayed green fluorescence, whereas when cells were incubated with fluorescent siRNA in the absence of a transfection reagent no fluorescent cells were seen. Nevertheless, it is possible that low levels of siRNA (below visual detection) were taken up by the cells and that these could have an effect. To test this, REE/CTVM28 cells were treated with siRNA targeting *RLuc* in the presence or absence of Lipofectamine 2000 and, at 24 h post-treatment, infected with SFV4-st*RLuc*. Luciferase levels were measured 48 h later. Luciferase levels in cells incubated with siRNA and a transfection reagent were significantly lower than those in non-transfected cells, confirming successful uptake of siRNA and silencing of the *RLuc* gene, while levels in cells that received the siRNA without a transfection reagent were unchanged indicating that significant uptake of siRNA had not occurred (Fig. 2a).

Having established that the two transfection reagents Lipofectamine 2000 and Xtreme facilitated introduction of siRNA into a proportion of cells for each of the tick cell lines without causing major deleterious effects, the ability of the two reagents to facilitate silencing of the expression of the *RLuc* virus reporter gene was tested. Nine tick cell lines were transfected using either transfection reagent with siRNA targeting *RLuc* or with a scrambled siRNA as a control and incubated for 24 h. The cells were then infected with SFV4-st*RLuc* and incubated for a further 48 h. Subsequently cells were lysed and the level of luciferase was measured. Figure 2b shows luciferase expression for all the cell lines. The majority of cell lines showed a reduction in luciferase levels when transfected with the siRNA, although the effectiveness varied according to the transfection reagent used. BME/CTVM23 cells for example did not show any change with Xtreme but showed a significant reduction with Lipofectamine 2000, while IDE8 and ISE6 cells showed similar reductions with both transfection reagents.

### The efficiency of long dsRNA uptake depends on the cell line and whether a transfection reagent is used

To examine how efficiently each of eight tick cell lines took up long dsRNA, fluorescent long dsRNA was made and added to cells in the presence or absence of the transfection reagents Lipofectamine 2000 or Xtreme. The eight cell lines were examined by fluorescence microscopy after 24 h. All the lines displayed an ability to take up long dsRNA in the absence of a transfection reagent although the efficiency of uptake varied greatly between different lines, with some showing almost no fluorescent dsRNA in cells (Table 4). Apart from in BME/CTVM23 and RAE/CTVM1, there were more fluorescent cells with Xtreme than with Lipofectamine 2000. Moreover, the amount of dsRNA taken up by individual cells appeared to increase when a transfection reagent was used. Representative confocal images of IDE8 cells transfected with fluorescent long dsRNA are shown in Fig. 1i–l.

**Table 4:** Efficiency of uptake of fluorescent long dsRNA by eight tick cell lines

	Percentage of cells containing detectable green fluorescence when incubated with dsRNA and:		
Cell Line	Lipofectamine 2000	Xtreme	No reagent
AVL/CTVM13	68	73	23
BDE/CTVM16	55	62	<2
BME/CTVM23	54	50	<2
HAE/CTVM9	65	79	<2
IRE/CTVM19	29	62	<2
IDE8	45	51	4
ISE6	53	77	<2
RAE/CTVM1	70	49	18

Cells were incubated with fluorescent long dsRNA either alone or with one of two transfection reagents, Lipofectamine 2000 or Xtreme (one tube of cells per treatment)

### Silencing of a foreign virus reporter protein can also be achieved using long dsRNA

**Fig. 3:**
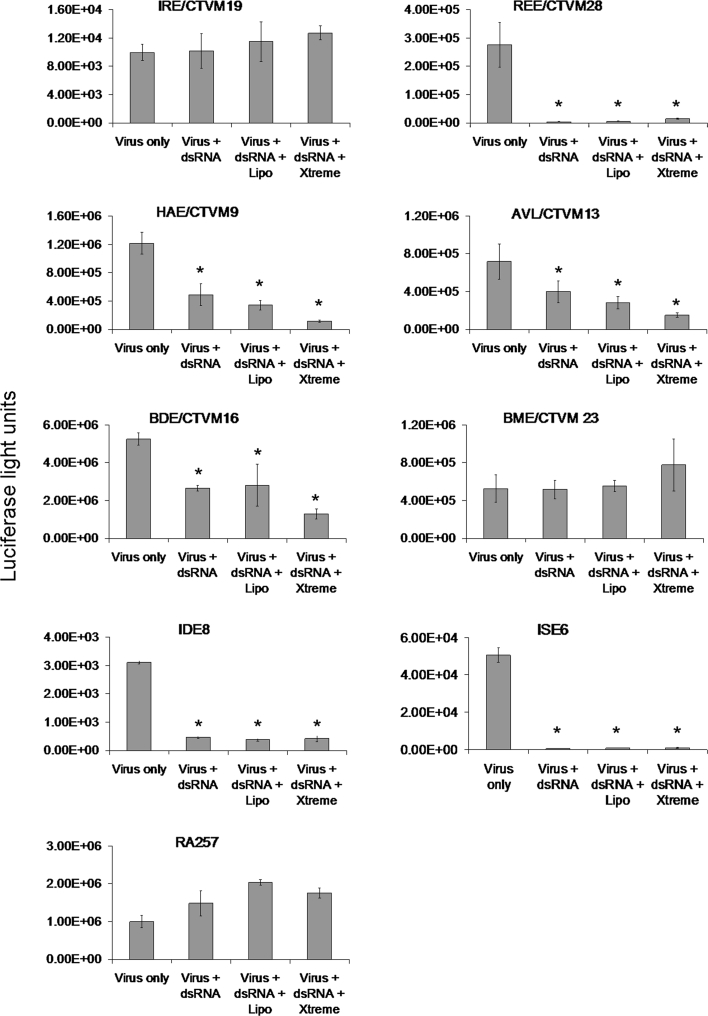
Silencing of a foreign virus reporter protein by long dsRNA treatment. Nine tick cell lines were incubated with long dsRNA targeting *RLuc* in the presence or absence of a transfection reagent and then infected 24 h later with SFV expressing *RLuc*. Luciferase levels (*y*-axis) were then measured 48 h later in cultures with SFV alone (Virus only), dsRNA followed by SFV (Virus + dsRNA), dsRNA with Lipofectamine 2000 followed by SFV (Lipo + dsRNA + virus) and dsRNA with Xtreme followed by SFV (Xtreme + dsRNA + virus). The values shown are means of four replicate cultures and the *error bars* are standard deviations of the mean. The scale used for each *y*-axis reflects the range of luciferase levels generated in the particular tick cell line. * Significant decrease from the virus only control

In many previous studies using dsRNA, gene silencing in tick cells was carried out by adding the long dsRNA directly to the tick cell cultures without the aid of a transfection reagent (Blouin et al. 2008; de la Fuente et al. 2007b, 2008; Zivkovic et al. 2010a). To determine whether or not the use of a transfection reagent would enhance the dsRNA silencing of a virus reporter gene, nine tick cell lines were incubated overnight with dsRNA against luciferase either alone or in the presence of one of the two transfection reagents Lipofectamine 2000 or Xtreme, then infected with SFV4-st*RLuc* and assayed for reduction in luciferase activity 48 h post-infection. As with the use of siRNA, the results varied greatly according to the cell line (Fig. 3). In some cell lines there was a dramatic silencing of reporter gene expression; in ISE6 cells for example the dsRNA significantly decreased expression by an average of 98.5 %. In other lines however the dsRNA treatment had little impact; in IRE/CTVM19 cells for example the luciferase levels were not significantly different between cells that were treated or not treated with dsRNA. The use of a transfection reagent enhanced silencing in some of the cell lines while its presence did not appear to make any difference in others. It even seemed to be detrimental to some; for example in the RA257 cell line, luciferase levels increased significantly compared to cells treated with the virus alone when either Lipofectamine 2000 or Xtreme was used along with the dsRNA. There was no significant difference between RA257 cells treated with virus only and when dsRNA was used without a transfection reagent.

Also, within cell lines the different reagents varied in their effectiveness. In HAE/CTVM9 cells for example, both Lipofectamine 2000 and Xtreme with dsRNA showed significant mean decreases in luciferase expression of 72 and 91 % respectively compared to cells infected with virus only, and the difference between the Lipofectamine 2000 and Xtreme treatments was also significant.

### The effectiveness of using long dsRNA to silence an endogenous gene differs between cell lines of the same tick species and is incubation time-dependent

While silencing of foreign protein expression plays an important role in the study of tick-pathogen interaction and provides an easy-to-study system, gene silencing by RNAi is also used to analyse the function of endogenous tick genes and the parameters for this could differ from those for virus-infected cells. To investigate this possibility two *I. scapularis* cell lines, IDE8 and ISE6, were selected for study based on the availability of an almost complete genome sequence for this species and therefore their applicability to gene expression knockdown studies. Long dsRNA was used because of the previous observation (Fig. 3) that long dsRNA was very effective at silencing a foreign reporter protein in both these cell lines, in both the presence and absence of a transfection reagent.

**Fig. 4:**
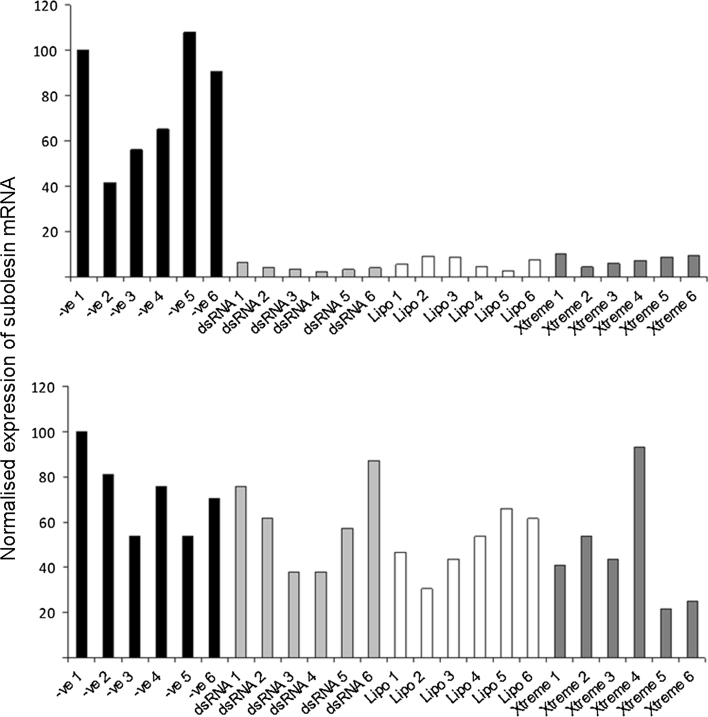
Efficiency of subolesin mRNA transcript knockdown in IDE8 and ISE6 cells. IDE8 (*upper*) and ISE6 (*lower*) cells were transfected (with or without a transfection reagent) with dsRNA targeting subolesin or negative control dsRNA targeting eGFP and incubated for 96 h. Data for all replicates are included to illustrate the level of variability encountered between individual cultures. Total RNA was extracted and equal amounts of RNA were used to make cDNA. Transcripts were measured by real-time PCR and C_t_ values for subolesin mRNA were then normalised against tick β-actin mRNA for each replicate. Subolesin mRNA expression levels (y-axis) are shown in arbitrary units. *–ve* dsRNA targeting eGFP, *dsRNA*  only dsRNA targeting subolesin, *lipo* Lipofectamine 2000 + dsRNA targeting subolesin, *x* Xtreme + dsRNA targeting subolesin

Firstly, both IDE8 and ISE6 cell lines were incubated with dsRNA targeting RNA from the endogenous gene subolesin, with or without a transfection reagent, and incubated for 96 h (six replicates for each treatment). RNA was extracted from the cells, total amounts of RNA were measured, equal amounts of RNA were reverse transcribed into cDNA and subolesin RNA transcript levels were then measured by real-time PCR. β-actin was used as a reference gene. Subolesin has been previously implicated in various functions including tick immunity and has been successfully silenced by RNAi in previous studies (de la Fuente et al. 2006; Zivkovic et al. 2010b). In IDE8 cells the knockdown was consistent between replicates and highly effective, while in ISE6 cells the knockdown was weak and highly variable with or without a transfection reagent (Fig. 4).

**Fig. 5:**
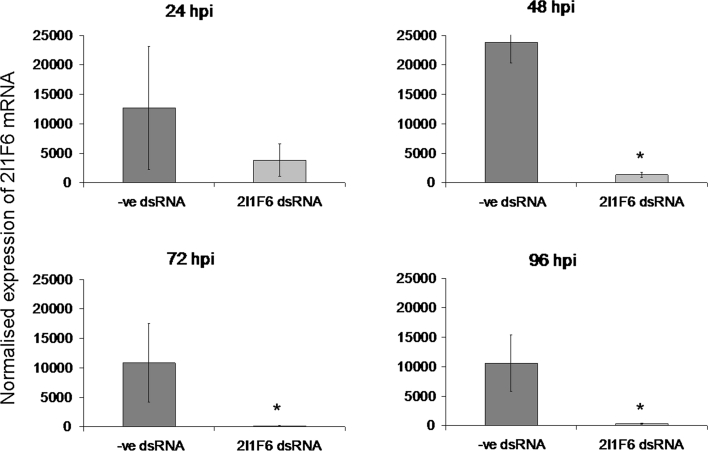
Efficiency of 2I1F6 mRNA transcript knockdown in IDE8 cells. IDE8 cells were transfected (without a transfection reagent) with dsRNA targeting 2I1F6 (2I1F6 dsRNA) or negative control dsRNA targeting eGFP (−ve dsRNA) and incubated for 24, 48, 72 or 96 h. Total RNA was extracted at each timepoint and equal amounts of RNA were used to make cDNA. Transcripts were measured by real-time PCR and C_t_ values for 2I1F6 mRNA were normalised against tick β-actin mRNA for each replicate. 2I1F6 mRNA expression levels (*y*-axis) are shown in arbitrary units. These values are means of four replicate cultures and the error bars are standard deviations from the mean. * significant difference between the −ve dsRNA and the 2I1F6 dsRNA

In previous studies tick cells were incubated with dsRNA for 3–5 days prior to further analysis (Blouin et al. 2008; Kurscheid et al. 2009; Zivkovic et al. 2010a). In RNAi experiments with mammalian and mosquito cells a shorter incubation time of 24–48 h is typical (Attarzadeh-Yazdi et al. 2009; Barry et al. 2010). It is important to make experiments both as effective and as efficient as possible so we sought to reduce the dsRNA incubation time for tick cells. Replicate cultures of IDE8 cells were transfected with dsRNA that targeted the protein 2I1F6 (hematopoietic stem/progenitor cells protein-like gene) (de la Fuente et al. 2007b) or negative control dsRNA targeting eGFP. RNA was then extracted from cultures 24, 48, 72 or 96 h later. Total amounts of RNA were measured and then equal amounts of RNA were reverse transcribed into cDNA. Levels of 2I1F6 transcript were quantified by real-time PCR to measure efficiency of knockdown. It was found that at 24 h post-transfection, the knockdown was inconsistent and not very effective. However, by 48 h post-transfection a dramatic and significant knockdown was achieved. This knockdown was sustained at 72 and 96 h post-transfection (Fig. 5).

## Discussion

In nature, RNAi plays a significant role in both plant and insect immune responses to virus infection by targeting the foreign dsRNA of virus genomes, or that which is created during the virus life cycle, for degradation (Ding and Voinnet 2007; Fragkoudis et al. 2009a). For example, in *Drosophila*, mutations in the RNAi pathway significantly increase susceptibility to *Drosophila* X virus infection (Zambon et al. 2006). Furthermore, the existence of plant- and insect-borne viruses with RNAi suppressor mechanisms illustrates how effective RNAi can be as a host defence mechanism (Bivalkar-Mehla et al. 2011; Song et al. 2011).

The use of RNAi as a crucial molecular tool has increased dramatically in recent years. It allows for the rapid knockdown of protein expression in vitro and in vivo and the consequent analysis of protein function. Ticks are known carriers of viruses, bacteria and protozoa that can infect and cause disease in vertebrates (Jongejan and Uilenberg 2004). Using RNAi as a tool to understand the interaction between ticks and pathogens may allow the development of new control measures to prevent the spread of these pathogens.

RNAi techniques have been used previously in both ticks and tick cells (Kocan et al. 2007; Nijhof et al. 2007; de la Fuente et al. 2007c; Kurscheid et al. 2009) although not as widely in tick cells as in mammalian or insect cells. This study was intended to investigate the effectiveness of RNAi in tick cells and to improve on existing RNAi protocols. When RNAi has been used previously, optimised conditions of use have not always been defined and inconsistency in knocking down the expression of different tick genes remains an issue.

To address this we assembled a panel of cell lines derived from eight different tick species within four ixodid genera of medical and veterinary importance. We included three of the cell lines which have been used previously for gene silencing by other research groups, namely IDE8, ISE6 and IRE/CTVM19 (de la Fuente et al. 2007b, 2008; Blouin et al. 2008; Kurtti et al. 2008; Pedra et al. 2010). We did not have access to the *R.* (*B.*) *microplus* cell line BME26 which has also been used previously (Kurscheid et al. 2009; Zivkovic et al. 2010a). Instead we selected a different *R.* (*B.*) *microplus* line BME/CTVM23, and the *R.* (*B.*) *decoloratus* line BDE/CTVM16. We also examined cell lines from four other economically important tick species: *A. variegatum* (AVL/CTVM13), *H. anatolicum* (HAE/CTVM9), *R. appendiculatus* (RA257 and RAE/CTVM1) and *R. evertsi* (REE/CTVM28).

The main priority of this study was to improve the efficiency and consistency of siRNA and long dsRNA use in tick cells in vitro. Various different parameters that can affect the successful use of RNAi to knock down mRNA levels and protein expression were examined for both siRNA and long dsRNA. Both can be used effectively in tick cells but different cell lines require different conditions.

Transfection reagents are commonly used to aid the transfection of siRNA or long dsRNA into cultured cells. Some cell types, however, do not require a transfection reagent as they naturally take up the RNA (Clemens et al. 2000; Lingor et al. 2004). The requirement for a transfection reagent in a panel of tick cell lines was tested using fluorescent siRNA and long dsRNA that could be seen microscopically. This method was also used in a smaller study carried out by Blouin et al. (2008) when they tracked the uptake of long dsRNA by IDE8 cells through the use of fluorescent long dsRNA. Previous observations that long dsRNAs do not require a transfection reagent for uptake were confirmed; fluorescent long dsRNA was detected in all the cell lines although the efficiency of uptake varied widely. In the case of siRNA, a transfection reagent was required and the efficiency of uptake was dependent on the transfection reagent used. While it was not feasible to test every commercially available transfection reagent, six commonly used reagents were selected; the most effective were Lipofectamine 2000 and Roche XtremeGene (Xtreme). Levels of transfection were calculated based on microscopic observational quantification. It must be noted however, that this system may have omitted cells that contained fluorescent RNA at a low level that could not be detected visually. This implies that actual transfection efficiencies may be higher than the figures obtained by visual examination as even low amounts of RNA may be effective at knocking down mRNA and protein expression.

Infection of cells with SFV-st*Rluc* was used to measure the ability of dsRNA or siRNA to knock down foreign protein expression in tick cells. Cells were transfected with siRNA (using Lipofectamine 2000 or Xtreme) or long dsRNA (with or without a transfection reagent) against *RLuc* and then infected with SFV-st*RLuc*. Both the long dsRNA and the siRNA treatments were effective. The levels of *RLuc* protein decreased significantly in many of the treated cultures. According to Garcia et al. (2005, 2006), the presence of virus-specific small RNAs in SFV-infected ISE6 cells indicates the induction of the exogenous RNAi pathway. Similar results have been found for viral infections in other organisms, including plants, *Drosophila* and mosquitoes (Donald et al. 2012). While the exogenous RNAi pathway is activated by viral infections, it has been shown in different arthropod cell systems that virus-specific dsRNA or siRNAs added to the culture are also able to be processed and target the virus through this pathway, resulting in silencing as observed in the present study with SFV-st*Rluc* infections. Although the endogenous RNAi pathway, believed to play a role in ensuring genome stability by repressing transposons, shares several features with the exogenous RNAi pathway, its possible role in viral infections has not been elucidated (Donald et al. 2012).

The amount of dsRNA used in previous studies varied from approximately 100 ng (Blouin et al. 2008) to 800 μg (Pedra et al. 2010) added to each well of a 24-well plate. In the present study, the concentration used (200 ng/well, 24-well plate) was an estimation based on pilot studies and previous experience with other types of cell lines as well as previous studies with tick cells. However, the concentration may need to be optimised further. In the single published study using siRNA in tick cells (Pedra et al. 2010), the authors introduced siRNA or long dsRNA into IRE/CTVM19 cells with the aid of a transfection reagent. They also used very high amounts of siRNA, as compared to other arthropod in vitro systems such as mosquito cells. In the present study, silencing of the reporter gene *RLuc* in SFV-infected IRE/CTVM19 cells using long dsRNA was negligible. siRNA however, was effective at a concentration 20,000 times lower than that used previously by Pedra et al. (2010).

Interestingly, when comparing the two types of nucleic acid in the same cell line, dsRNA appeared to be more efficient than siRNA as it produced a greater knockdown in *RLuc* expression in the majority of the cell lines tested. This was despite using approximately 10 times more siRNA than dsRNA. While the siRNA used was a pool of different sequences targeting different regions of the *RLuc* gene with the aim of enhancing effectiveness, introducing siRNAs bypasses two important steps in the RNAi pathway that may influence the efficiency of a knockdown. Long dsRNA is normally bound and cleaved into siRNAs by a protein called Dicer. These siRNAs are then incorporated into a RISC complex that is involved in targeting RNAs that correspond to the siRNA and cleaving them (Hammond et al. 2000; Hannon 2002; Sifuentes-Romero et al. 2011). Not activating Dicer for example could dampen the RNAi response. It is also possible that long dsRNA, when cleaved by Dicer, produces a vast array of siRNAs that target numerous areas of the target gene thus making it more efficient. It must be remembered however that the use of long dsRNA can increase the likelihood of off-target effects and this should be taken into account (Lew-Tabor et al. 2011).

All of the cell lines tested took up dsRNA, as shown by the use of fluorescent long dsRNA. However, in the virus reporter gene silencing experiments, some of the cell lines (BME/CTVM23, RA257 and IRE/CTVM19) did not respond to dsRNA despite responding to siRNA. This suggests that these cells may be unable to process long dsRNA efficiently or correctly, possibly through a loss of Dicer functionality similar to that seen in the mosquito cell line C6/36 (Brackney et al. 2010; Morazzani et al. 2012; Scott et al. 2010). This may explain the need for such large amounts of dsRNA in the study of Pedra et al. (2010). We recommend the use of siRNAs for knockdown experiments with these cells.

Because long dsRNA appeared to be more efficient than siRNA at knocking down virus protein expression in some cell lines, long dsRNAs were used to test other variables such as cell type and incubation time when trying to knock down an endogenous gene. The two genes selected, subolesin and 2I1F6 hematopoietic stem/progenitor cells protein-like, have both been successfully silenced previously in *I. scapularis* cells (Blouin et al. 2008; de la Fuente et al. 2007b). The knockdown of subolesin expression was consistent and very efficient with long dsRNA in IDE8 cells but showed wide variability and poor levels of knockdown in ISE6 cells despite the dsRNA being generated from ISE6 cells. All the replicates were shown in Fig. 4 to demonstrate the level of variability encountered. This illustrates one of the inherent features of work thus far with tick cell lines; knockdown efficiency can vary dramatically depending on the cell line used. ISE6 and IDE8 are both *I. scapularis* cell lines but they responded quite differently to long dsRNA. Moreover, we found that, despite good uptake of fluorescent long dsRNA as determined by fluorescence microscopy, silencing of the virus reporter gene in the *R.* (*B.*) *microplus* cell line BME/CTVM23 was negligible with long dsRNA and poor with siRNA, even when a transfection reagent was used. In contrast, using dsRNA in the cell line BME26, derived from the same tick species, Zivkovic et al. (2010a) reported 88.1 % silencing of the subolesin gene and between 60.5 and 99.5 % silencing of other tick genes and Kurscheid et al. (2009) achieved between 31 and 100 % knockdown of a panel of ten tick genes.

In previous studies, the effect of long dsRNA on tick cells was measured after incubation for between 72 and 120 h (Blouin et al. 2008; de la Fuente et al. 2008; Kurscheid et al. 2009). In the present study it was found that at 24 h post-transfection with 2I1F6-specific dsRNA, 2I1F6 RNA levels were beginning to decrease but this was highly variable and inconsistent. However, by 48 h the knockdown was very high and consistently reproducible. This knockdown was achieved without the use of a transfection reagent although our fluorescence microscopy experiments suggested that very few IDE8 cells were taking up long dsRNA. The high level of endogenous gene knockdown indicates that long dsRNA does indeed enter the majority of cells but at concentrations below the level of detection by fluorescence microscopy.

Overall, our results indicate the importance of selecting the most suitable tick cell line for the intended research. While conditions for different cell lines varied, parameters have been established that can be followed and can consistently produce results. The methods described in this study provide a template that can be followed and adapted according to requirements as research with different tick cell lines increases.
